# Advance of Molecular Imaging Technology and Targeted Imaging Agent in Imaging and Therapy

**DOI:** 10.1155/2014/819324

**Published:** 2014-02-13

**Authors:** Zhi-Yi Chen, Yi-Xiang Wang, Yan Lin, Jin-Shan Zhang, Feng Yang, Qiu-Lan Zhou, Yang-Ying Liao

**Affiliations:** ^1^Department of Ultrasound Medicine, Laboratory of Ultrasound Molecular Imaging, The Third Affiliated Hospital of Guangzhou Medical University, Guangzhou 510150, China; ^2^Department of Imaging and Interventional Radiology, Prince of Wales Hospital, The Chinese University of Hong Kong, Shatin, New Territories, Hong Kong; ^3^Department of Nuclear Medicine, The Third Affiliated Hospital of Guangzhou Medical University, Guangzhou 510150, China

## Abstract

Molecular imaging is an emerging field that integrates advanced imaging technology with cellular and molecular biology. It can realize noninvasive and real time visualization, measurement of physiological or pathological process in the living organism at the cellular and molecular level, providing an effective method of information acquiring for diagnosis, therapy, and drug development and evaluating treatment of efficacy. Molecular imaging requires high resolution and high sensitive instruments and specific imaging agents that link the imaging signal with molecular event. Recently, the application of new emerging chemical technology and nanotechnology has stimulated the development of imaging agents. Nanoparticles modified with small molecule, peptide, antibody, and aptamer have been extensively applied for preclinical studies. Therapeutic drug or gene is incorporated into nanoparticles to construct multifunctional imaging agents which allow for theranostic applications. In this review, we will discuss the characteristics of molecular imaging, the novel imaging agent including targeted imaging agent and multifunctional imaging agent, as well as cite some examples of their application in molecular imaging and therapy.

## 1. Introduction

Molecular imaging is a rapidly developed multidiscipline which involves molecular biology, chemistry, computer, engineering, and medicine [1]. It can realize noninvasive and real time visualization, measurement of physiological or pathological process in the living organism at the cellular or molecular level [2, 3]. And it also allows repeated studies in the same animal, thus making it possible to collect longitudinal data and reduce the number of animals and cost [4]. Therefore, molecular imaging plays an important role in earlier detection, accurate diagnosis, and drug development and discovery [5–7]. Molecular imaging requires high resolution and high sensitive instruments to detect specific imaging agents that link the imaging signal with molecular event [8]. There are five imaging modalities available for molecular imaging, including X-ray computed tomography imaging (CT), optical imaging (OI), radionuclide imaging (involving PET and SPECT), ultrasound (US) imaging and magnetic resonance imaging (MRI) [9]. In the past two decades, imaging instruments have grown exponentially. Improvement in instruments and iterative image reconstruction has resulted in high resolution images that reveal tiny lesion and realize accurate quantification of biological process. A parallel development has been the preparation of imaging agents which can bind their targets with high specificity and affinity [10]. In this review, we will discuss the characteristics of molecular imaging, some novel imaging agents based on nanoparticles including targeted imaging agent and multifunctional imaging agents, and cite some examples of their application in molecular imaging and therapy.

## 2. Molecular Imaging Technology

### 2.1. Radionuclide Imaging

Radionuclide molecular imaging including PET and SPECT is the earliest and most mature molecular imaging technique. Due to its advantages of high sensitivity and quantifiability, radionuclide molecular imaging plays an important role in clinical and preclinical researches [11]. Over the past decade, with the progress of molecular biology and radiochemistry, a variety of tracer with high specificity and affinity appeared. A lot of preclinical and clinical studies have confirmed the feasibility of using radionuclide molecular imaging to detect tumor and predict response to therapy [12, 13].

#### 2.1.1. PET

PET is the molecular imaging modality most extensively used in current clinic routine. It measures the signal originated from the radioactive decay of neutron-deficient radioisotopes (such as ^11^C, ^15^O, ^18^F, and ^131^I) that are intravenously injected into the body. These isotopes emit positrons which are ejected from the nucleus as a result of springless interactions with electrons in surrounding tissue. The positrons rapidly lose kinetic energy by spreading around the tissue and collide with an electron to form two 511 keV photons which are taking trajectory 180° apart, and this is an event known as annihilation [14]. A PET detector surrounding the subject is designed to detect the signal and convert the resulting electrical signal into sinograms that are finally rebuilt into tomographic images.

Because of its high sensitivity of 10^−11^~10^−12^ mol/L, limitless depth of penetration, and quantitative capabilities, PET becomes a powerful tool for clinical diagnosis and basic research including neurology, cardiology, and particularly oncology [15, 16]. In the clinic, PET is crucial for cancer detection and staging, as well as evaluation of response to therapy. A mass of radiotracer has been employed for cancer imaging, with ^18^F-FDG being the key one. The main disadvantage of PET is the lack of anatomical parameters to identify molecular events with accurate correlation to anatomical findings, and this disadvantage has recently been compensated by merging these devices with either CT or MRI [17]. It is reported that whole-body PET/CT improves the accuracy of cancer diagnosis and staging. With the widespread of instrument, PET/CT has become an important tool for predicting therapeutic response, providing useful information for the decision to stop ineffective treatment or switch to a more efficient treatment. It is shown that up to 40% of patients with cancer have changed the treatment because of application of PET/CT [18]. Recently, Andrade et al. [19] evaluated 40 patients with invasive ductal breast carcinomas that received neoadjuvant chemotherapy by FDG-PET/CT. They found that the application of FDG-PET/CT after the second course of NAC could predict therapeutic response in ductal breast carcinomas and potentially recognise the nonresponding patients for whom ineffective chemotherapy should be avoided. Other limitations of PET are their safety profile and the requirement of cyclotron to produce radiopharmaceuticals.

#### 2.1.2. SPECT

**Figure 1:**
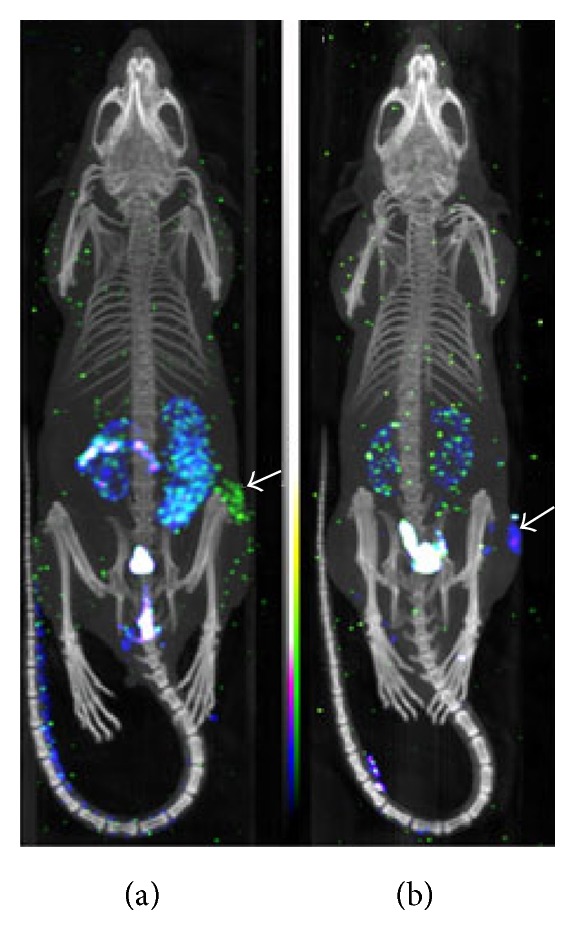
Dual-isotope SPECT/CT images of tumor-bearing mice. (a) The mice were imaged 1 h after coinjection of 1.32 nmol ^177^Lu—DOTA—cRGDfK (yellow-green colormap) and 1.32 nmol ^111^In—DOTA—cRADfK (blue-purple colormap); (b) the mice were imaged 1 h after coinjection of 1.32 nmol ^111^In—DOTA—cRGDfK (blue-purple colormap) and 1.32 nmol ^177^Lu—DOTA—cRADfK (yellow-green colormap). The white arrows indicate the locations of tumor [23].

Unlike PET, SPECT directly detects gamma-ray photon emitted by the chosen radionuclide during their decay. Compared with PET, SPECT is more affordable and extensively employed in the clinical routine, but it is generally less sensitive since the photons which are not traveling along the axis of the collimator are rejected by the scanner. The spatial resolution (8–10 mm) of SPECT is lower than that of clinical PET (5–7 mm) [20], but the spatial resolution of small animal SPECT (micro-SPECT) is higher than that of PET due to the development of imaging equipment. Thus micro-SPECT is more available in preclinical investigations including the transformation research and animal studies such as oncology, neurology, cardiovascular disease, and drug development [21, 22]. Additionally, since the radionuclides commonly available for SPECT have longer half-life periods (ranging from a few hours to days), longitudinal studies can be performed. Based on the isotope-specific energies of the emitted photons (e.g., [^111^In] indium: 171 and 245 keV; [^177^Lu] lutetium: 202 and 307 keV), SPECT can distinguish different radioisotopes, therefore making it possible to image different targets simultaneously. Hijnen et al. [23] took advantage of this characteristic and devised a dual-isotope experiment on a micro-SPECT system. In their study, they quantified the biodistribution and tumor uptake of the angiogenesis tracer cRGD via SPECT (Figure 1). However, dual-tracer images can be severely distorted due to cross talk between the two isotopes. Recently, Hapdey et al. [24] put forward a generalized spectral factor analysis (GSFA) method for solving this problem in simultaneous ^99m^Tc/^123^I SPECT, which proved that simultaneous ^99m^Tc/^123^I imaging can provide images of similar quantitative accuracy through GSFA as when using sequential and scatter-free^99m^Tc/^123^I imaging in brain SPECT.

### 2.2. Magnetic Resonance Imaging

Magnetic resonance imaging (MRI) is a highly versatile imaging modality [25]. During the past decades, improvement in instrument launched the field of MRI into a new era of molecular imaging. The merits of MRI as an imaging modality for molecular imaging are relatively high temporal and spatial resolution, excellent tissue contrast and tissue penetration, no ionizing radiation, noninvasiveness for serial studies, and simultaneous acquisition of anatomical structure and physiological function [26]. Nevertheless, molecular MRI is limited by its relatively low sensitivity, and this requires the introduction of imaging agent and development of powerful signal amplification strategies. Imaging agent design is hence an important topic in molecular MRI. Currently, the MR imaging agents are mainly divided into two kinds: ferromagnetism contrast agents and paramagnetic contrast agents. The former is considered as negative contrast agents which mainly reduce the signal in T2-weighted images, while the latter is referred to as positive contrast agents that increase the signal in T1-weighted images. The most representative negative contrast agents are superparamagnetic iron oxide (SPIO) and ultrasmall superparamagnetic iron oxide (USPIO), and typical positive contrast agents are small molecular weight compounds involving a single Lanthanide chelate as signal producing element (e.g., gadolinium-DTPA). To date, there are numerous examples of MR molecular imaging which have confirmed the potential of this technology. Rapley et al. [27] demonstrated that the application of antibody-SPIO complex and MRI was a feasible method for detecting and measuring nucleosome concentration in vitro, which was expected to become a diagnostic, prognostic, and predictive tool in the management of cancer. Recently, Debergh et al. [28] showed that the application of a small monogadolinated tracer targeting *α*_v_ *β*_3_ integrin and MR molecular imaging was a promising strategy for evaluation of colorectal cancers associated angiogenesis.

### 2.3. X-Ray Computed Tomography Imaging

**Figure 2:**
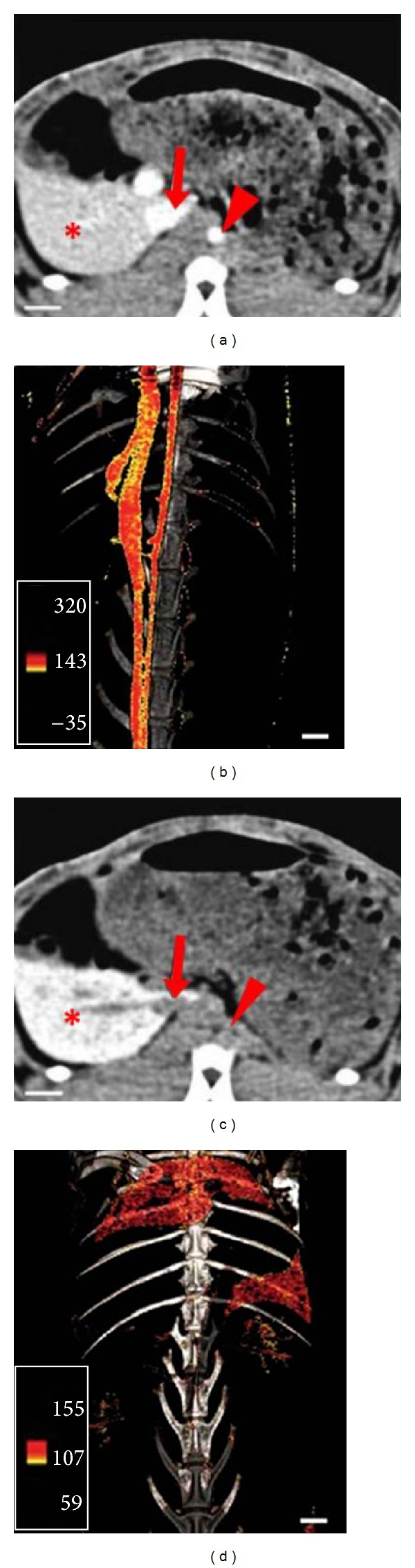
CT molecular imaging based on iodinated nanoparticles (N1177). The kinetics and distribution of iodinated nanoparticles (N1177) in the atherosclerotic rabbit model were displayed by CT imaging. (a) 5 min after intravenous injection of N1177, CT image display aorta, and vena cava (red arrow) with a noticeable signal; (b) the reconstruction of three-dimensional CT imaging; (c) 2 h after intravenous injection of N1177, a strong signal was displayed in the spleen (∗); (d) use of color scale to reconstruct three-dimensional CT angiograms of CT scan. (adopted from Hyafil et al. [33]).

CT imaging technologies have undergone a very fast development in the last years. High resolution small animal CT (micro-CT) has transformed CT imaging from organ, tissue to molecular level, which is playing an increasingly important role in preclinical researches [29, 30]. The main advantages of CT are high spatial resolution (micro-CT is 0.02~0.30 mm; clinical CT is 0.5~2.0 mm), fast acquisition time, relative simplicity, availability, excellent hard-tissue imaging. Due to limitations like ionizing radiation, limited soft tissue resolution, and poor sensitivity (10^−2^~10^−3 ^mol/L), CT is always combined with other imaging modalities such as SPECT, PET to provide anatomical parameters for the biochemical and physiological findings [31]. Recently, the development of CT contrast agents has brought new hope for CT molecular imaging [32]. For example, Hyafil et al. [33] reported cellular imaging of macrophage in the atherosclerotic plaque in a rabbit model through CT with iodinated nanoparticles (N1177) (Figure 2). Pan et al. [34] found that polymeric nanoparticles contrast agents which were comprised of organometallics or radiopaque organically soluble elements could further improve the imaging sensitivity for CT. Also, targeted gold nanoparticles for CT imaging at the cellular and molecular level have been successfully prepared. Li et al. [35] conjugated 2-deoxy-D-glucose (2-DG) to a gold nanoparticle to prepare CT molecular imaging agent (AuNP-2-DG) which could be used for CT imaging to obtain high resolution anatomic structure and metabolic information of tumor. The results of in vitro experiments proved that AuNP-2-DG could be used as a functional CT contrast agent. However, though the findings from these CT molecular imaging experiments show attractive prospect, further study is required to explore the feasibility of CT molecular imaging.

### 2.4. Optical Imaging

Optical molecular imaging technology is an emerging technology, based on genomics, proteomics, and modern optical technology. At present, the most widely used optical molecular imaging modalities in vivo include bioluminescence imaging (BLI) and fluorescence imaging. As optical imaging is performed through the use of light, thus it is considered as relatively safe. And due to their advantages of high sensitivity and low cost, optical imaging plays a central role in the investigation of tumor occurrence, progressions and relevant drug development [36, 37]. The main disadvantages of optical imaging are that the depth of penetration is limited as the energy of photons is low, thereby making it nearly impossible to image deep tissues in large subjects. Due to the small size of experimental animals, the depth of penetration is not a problem for them. Therefore, optical imaging is widely used for preclinical research.

**Figure 3:**
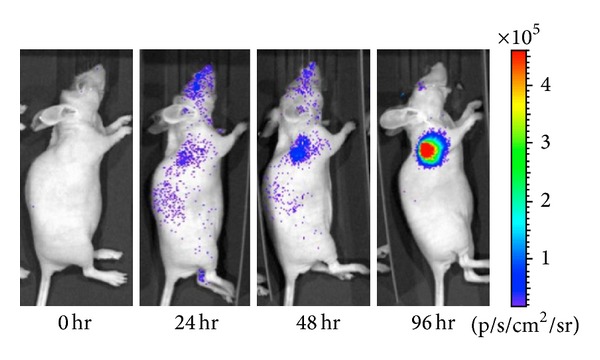
After doxorubicin treatment (5 mg/kg), bioluminescence imaging was performed to observe caspase activation in 22B-pcFluc-DEVD (adopted from Niu et al. [38]).

BLI is taking advantage of the light produced by the enzymatic oxidation reaction of luciferase and its substrate. The luciferase is usually generated from the reporter gene. As opposed to fluorescence imaging, the light source of BLI is from interior. And since tissue do not produce endogenous bioluminescence, the sensitivity and signal to background is better than fluorescence imaging. BLI is widely used to observe the biological processes in vivo like disease progression, certain gene expression, tumor growth, and metastasis and evaluate the effect of certain treatments. For example, Niu et al. [38] introduced a caspase-3 specific cyclic firefly luciferase reporter gene (pcFluc-DEVD) into UM-SCC-22B and 4T1 cells then induced apoptosis of the cells by different concentrations of doxorubicin and evaluated the effect by BLI. The results exhibited that BLI intensity was increased as early as 24 h after treatment and achieved a maximum at around day 12, which indicated that BLI with pcFluc-DEVD as a reporter gene can help to observe the kinetics of the apoptotic process in a real-time manner (Figure 3).

**Figure 4:**
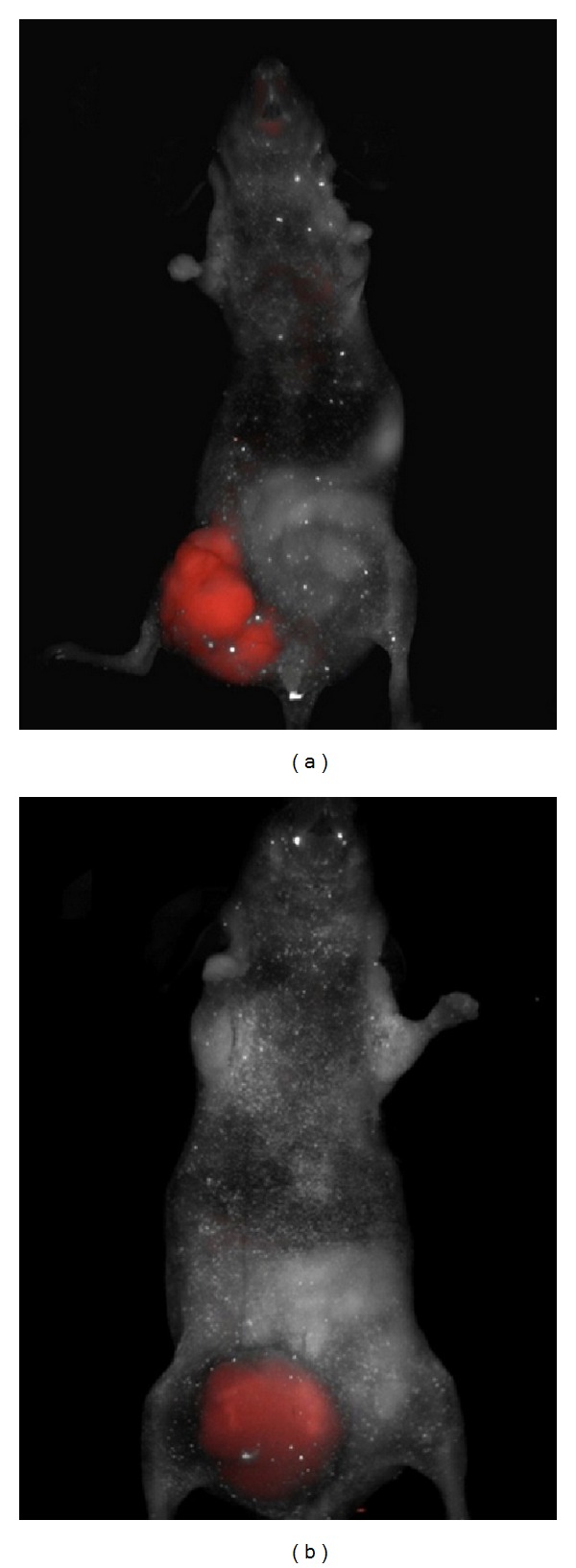
Nude mice was injected with SW620 CRC cells then administered intravenously with integrin *α*_v_ *β*_3_ fluorescent probe after the formation of intestinal tumor. Via a fluorescent scanner, the targeted fluorescence signals for *α*_v_ *β*_3_ were observed in vivo (a). A fluorescent-labeled antibody against the VEGFR was injected intravenously, and fluorescence was detected using a multispectral in vivo imaging system (b) (adopted from Atreya et al. [39]).

Fluorescence imaging is a versatile technique with a number of strengths such as relative low cost and multiplexed imaging. As light is absorbed by hemoglobin and other molecules, the depth of penetration is limited (usually <1 cm). Hence, fluorescence imaging is mainly used in animal researches. Atreya et al. [39] took advantage of the feature that integrin *α*_v_ *β*_3_ and vascular endothelial growth factor receptor (VEGFR) are overexpressed in tumor; they used the integrin *α*_v_ *β*_3_ fluorescent probe to detect the tumor angiogenesis in nude mice. The result showed there were strong fluorescence signals for *α*_v_ *β*_3_ in vivo via a fluorescent scanner, which indicated angiogenesis increased in the cancer tissue. Also, they proved that the targeted visualization of VEGF could be achieved via a fluorescent-labeled antibody against the VEGFR (Figure 4).

### 2.5. Ultrasound Molecular Imaging

With the use of ultrasound contrast agent, ultrasound imaging enables specific and sensitive depiction of molecular targets [40, 41]. Compared with other molecular imaging modalities, ultrasound molecular imaging has many advantages including good temporal resolution, quantitative data, real-time practice, noninvasiveness, relatively inexpensive cost, and no ionizing radiation. In addition, it is a unique modality in some sense that it can be employed for diagnostic imaging and as a therapeutic tool [42, 43]. Mancini et al. [44] proved that the use of ultrasound molecular imaging with microbubble targeting VEGFR-2 might be a valuable method for noninvasively detecting and quantifying of VEGFR-2 expression in thyroid cancer in mice, and it is also a useful tool for differentiating benign from malignant thyroid nodules.

Besides, for diagnostic purposes, molecular ultrasound imaging can also be applied for theranostic purposes. This is mainly achieved by loading gene or drug onto microbubble. The interaction between ultrasound and microbubble results in stable and inertial cavitation, which can transiently increase vascular and cellular membrane permeability, thereby potentially augments the extravasation and/or take up of drugs or genes loaded on microbubble. Li et al. [45] prepared a novel microbubble carrying 10-HCPT, and they demonstrated that the injection of 10-HCPT loaded microbubble and exposure to ultrasound could increase the drug concentration in tumor remarkably, leading to a significant increase in tumor inhibition rate (70.6%) compared with 10-HCPT loaded microbubble alone (47.8%) as well as commercial HCPT injection (49.4%). Our studies have confirmed that the combination of ultrasound and microbubble could significantly increase the transfection efficacy [46].

### 2.6. Multimodality Imaging

Among all molecular imaging techniques, every molecular imaging technique has its advantages and disadvantages. No single one is perfect and enough to provide comprehensive information for disease diagnosis [47]. In general, CT, MRI, and US are anatomic imaging methods but they have low sensitivity. Radionuclide imaging and optical imaging are functional imaging techniques, while they suffer from low resolution, which often lack structural parameter. The combination of different molecular imaging techniques, namely, multimodality imaging, can provide synergistic advantages over any modality alone and compensate for the disadvantages of each imaging system while taking advantage of their individual strengths, which has become the developmental trend of modern medical image now [48–51]. Multimodality imaging such as PET/SPECT, PET/CT, and PET/MRI can be used to obtain anatomical and molecular information while providing enough information for clinical diagnosis [52]. For example, the combination of PET and CT can produce coregistered data providing regions of increased ^18^F-FDG accumulation on the PET image with precise correlation to anatomical findings' locations on the CT scan, thus the specificity and sensitivity of PET in detecting lesion are enhanced, so as the accuracy in delineating target volume. Abgral et al. [53] conducted a study that included 91 patients who once had HN cancer but were cured and without any clinical evidence of recurrence to assess the diagnostic capabilities of ^18^F-FDG PET/CT in these patients. The results showed that the sensitivity, specificity, and accuracy of ^18^F-FDG PET/CT for the diagnosis of HN cancer recurrence were 100%, 85%, and 90%, respectively. The positive predictive value was 77%, while the negative predictive value was 100%.

**Figure 5:**
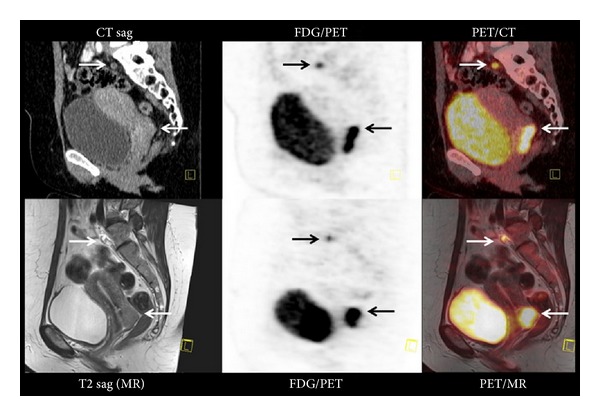
31-year-old female patient with cervical cancer had an ^18^F FDG-PET/CT and PET/MRI examination for restaging following radiotherapy. PET/CT showed the primary tumour in the cervix behind the urine bladder and a lymph node in the pelvis, both indicated with arrows. PET/MR exhibited the same findings but with a more precise definition of the primary tumor (adopted from Kjær et al. [55]).

MRI shows obvious advantages over CT including excellent soft tissue contrast, high spatial resolution, and no ionizing radiation; thus studies of PET/MRI become the focus of concern and have achieved initial progress. Lee et al. [54] used PET/MRI to evaluate the presence of monocytes in nonischemic remote zone after myocardial infarction, which proved that inflammatory cells of infarct zone and distant noninfarcted area could be observed by PET/MRI images. Recently, PET/MRI has been applied to clinic. Kjær et al. [55] performed a PET/MRI examination on a female patient with cervical cancer for restaging following radiotherapy and compared the results with PET/CT, which showed that PET/MRI performed well compared to PET/CT. It had a more precise definition of the primary tumor (Figure 5). However, PET with MRI in a single gantry which is dedicated to simultaneous PET/MRI is technically more challenging.

In addition to the combination of PET and CT or MRI, other multimodality imaging methods have been reported, such as OI/US, OI/CT, OI/MRI, and US/MRI [56–58]. OI is a versatile technique in preclinical research as it possesses the advantages of high specificity and sensitivity, low cost, relatively high-flux, and convenience. Ultrasound, on the other hand, has strength of high resolution. The strengths of these imaging techniques are complementary. The combination of them, which takes advantage of high sensitivity of optical imaging and high resolution of ultrasound, may produce synergistic effect and provide images with good spatial and temporal resolution. Lately, Li et al. [56] designed a system which integrated 3D ultrasound imaging and 3D continuous transillumination fluorescence tomography. They evaluated the feasibility and performance of the system by using phantoms and atherosclerosis disease mouse model. Results proved that ultrasound structural images could improve the quality of the fluorescent image reconstruction and the activity of VCAM could be detected through the system, which showed a great potential for the application in in vivo molecular imaging. As this system could provide structural information while keeping the merits of low cost of fluorescence imaging, it is worthy of promotion and application for the future.

Therefore, it can be seen that multimodality imaging is not a simple addition of various imaging technologies but rather for producing synergistic effect. It can provide more information to understand the biological processes comprehensively and objectively. But it is still difficult to carry out multimodality imaging due to existing problems regarding the accuracy of coregistered image, extra ionizing radiation, the extra dosage of contrast agent, and the toxicity of fused contrast agents. Therefore, it is in urgent need of developing multimodality molecular imaging agent.

## 3. Imaging Agents

Molecular imaging depends greatly on the development of specific and sensitive imaging agents, which is a pivotal rate-limiting step in the development of molecular imaging [8]. In a molecular imaging study, imaging agents are mainly used for interrogating or coupling back about a specific target of interest [10]. They usually consist of signal component and targeting component. In recent years, the advancement of biochemistry has been achieved and the development of molecular imaging technologies has led to the production of a mass of molecular imaging agents [57]. A mass of targeting moieties, such as small molecule, peptide, antibody, and aptamer, is applied to decorate ligand-directed (“targeted”) imaging agents to recognise specific pathological tissues [9]. Furthermore, nanoparticles with unique properties have emerged as a promising class of molecule imaging agents. Targeting moieties, therapeutic drug or gene, and different imaging labels can be incorporated into nanoparticles to construct targeted imaging agents and multifunctional imaging agents which allow for multimodal imaging and theranostic applications [58, 59].

### 3.1. Targeted Molecular Imaging Agents Based on Small Molecules

**Figure 6:**
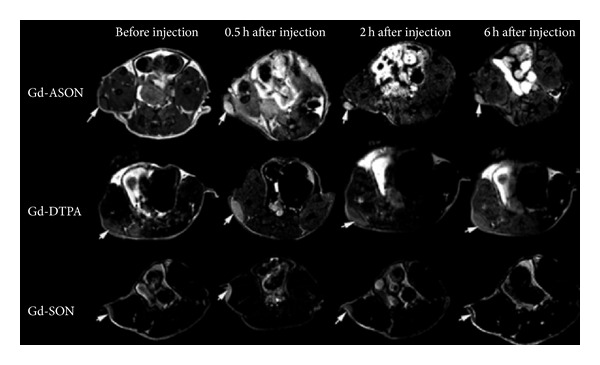
T1-weighted multiple slice multiple echo weighted MRI of nude mice bearing A549 tumors before and 0.5, 2, and 6 h after intraperitoneal injection. Tumors (arrows) all showed different enhancement level at 0.5 h, but in Gd-DOTA-ASON (upper line), the enhancement remained at 6 h, while Gd-DTPA (middle line) decreased obviously from 2 h (repetition time, 561 ms, echo time, 14 ms, and field of view, 4 cm); Gd-DOTA-SON (lower line) showed a similar trend to that of Gd-DOTA-ASON but with lower enhancement (adopted from Ren et al. [61]).

Small molecules, of which the size is usually less than 500 Da, are playing an important role in molecular imaging. Due to their small size, small molecules have a wide range of application including intracellular and central nervous system. ^18^F-FDG is the most widely used imaging agent based on small molecules, which is clinically applied for cancer imaging [60]. Ren et al. [61] reported the use of gadolinium (Gd) to label uniformly phosphorothioate-modified human tumor telomerase reverse transcriptase (hTERT) antisense oligonucleotide (ASON) targeting hTERT mRNA and conducted a study in BALB/c nude mice with 7.0T Micro-MRI, showing that Gd-DOTA-ASON was a potential intracellular MR contrast probe for detection of telomerase-positive carcinomas (Figure 6).

### 3.2. Targeted Molecular Imaging Agents Based on Peptide

Peptide is an important class of ligand used for molecular imaging [62]. Compared to small molecules, peptide has many advantages, such as superior selectivity and specificity and easier modification without changing their binding properties or distribution. Additionally, peptide is more stable in atmospheric temperature than antibodies and has a lower immunogenicity compared with antibodies. Recently, Hansen et al. [63] conjugated a peptide which could bind to the urokinase plasminogen activator receptor (uPAR) with high specificity and affinity to polyethylene glycol (PEG) coated USPIO nanoparticles. In vitro result showed that the peptide conjugated USPIO nanoparticles had a five times higher uptake in a uPAR positive cell line compared to the nanoparticles with a nonspecific peptide. Hackel et al. [64] used ^18^F labelled 2 cystine knot peptides and performed micro-PET on BxPC3 pancreatic adenocarcinoma xenografts in mice with them. Results indicated that these cystine knot peptide tracers had high tumor uptake, showing translational promise for cancer imaging.

### 3.3. Targeted Molecular Imaging Agents Based on Antibodies

**Figure 7:**
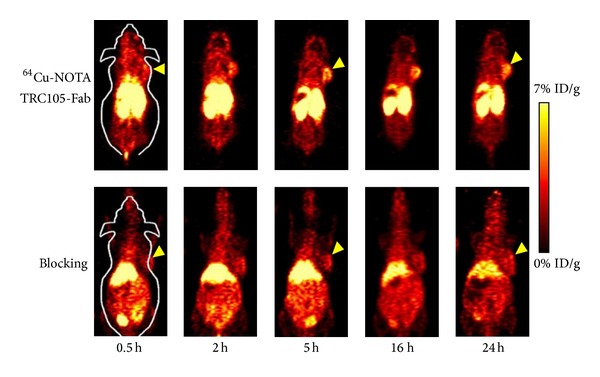
PET images were performed at 0.5, 2, 5, 16, and 24 h p.i. of ^64^Cu-NOTA-TRC105-Fab, or ^64^Cu-NOTA-TRC105-Fab after treatment with a 2 mg blocking dose of TRC105 before injection (adopted from Zhang et al. [66]).

Characterized by the ability to bind their target with high specificity and affinity and easy synthesis, antibodies have been applied for diagnosis and therapy [65]. So far, there are more than eight FDA-approved radiolabeled antibodies approved for SPECT molecular imaging and about twenty antibodies approved for therapy. Recently, Zhang et al. [66] reported the use of PET with (61/64) Cu-NOTA-TRC105-Fab to image CD105 expression, which showed obvious and target-specific uptake in the 4T1 tumor (Figure 7). Abdolahi et al. [67] conjugated superparamagnetic iron oxide nanoparticles with an antibody which could bind to the extracellular domain of PSMA and performed MRI with it. Results demonstrated that the nanoprobe could act as a specific MRI contrast agent for detection of PSMA-expressing prostate cancer cells.

### 3.4. Targeted Molecular Imaging Agents Based on Aptamer

**Figure 8:**
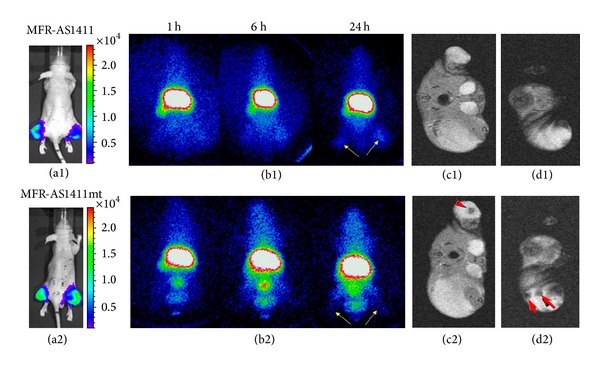
Cancer-targeted multimodality imaging through MFRAS1411 conjugated nanoparticles. Tumor-bearing mice were intravenously injected with MFR-AS1411 nanoparticles (b1) and MFR-AS1411mt (b2) nanoparticles (control group). Radionuclide images were performed at 1, 6, and 24 h after injection. Scintigraphic images of tumors in mice injected with MFRAS1411 exhibited that MFR-AS1411 nanoparticles were accumulated in the tumors but MFRAS1411mt were not. Tumor growth patterns were followed using bioluminescence signals acquired from luciferase-expressing C6 cells (a1, a2). MR images of tumor-bearing mice before (c1, d1) and after (c2, d2) injection of MFR-AS1411 were obtained. Dark signal intensities at tumor sites were detected in MFR-AS1411-injected mice (arrowhead) (adopted from Hwang et al. [73]).

Aptamer, single-stranded DNA or RNA oligonucleotides, can bind to their target with high selectivity and specificity [68]. Because they enjoy a number of merits including high affinity and specificity, low immunogenicity, small size, stable structures, and ease of production, aptamers have recently attracted increasing attention [69, 70]. In recent years, probe based on aptamer provides a new strategy for molecular imaging. Shi et al. [71] reported the use of an activatable aptamer probe (AAP) which could bind membrane proteins of living cancer cells with a high degree of specificity to visualize the cancer inside mice. The results showed that the AAP could be used to specifically, quickly detect CCRF-CEM cell in the serum or CCRF-CEM cell tumor in mice. Bagalkot et al. [72] recently described the use of a novel quantum dot conjugated with prostate specific membrane antigen (PSMA) RNA aptamers to image and cure cancer. Aptamer conjugated nanoparticles provide new strategy for targeted multimodality imaging. Hwang et al. [73] described a multimodal cancer-targeted imaging system for simultaneous fluorescence imaging, radionuclide imaging, and MRI in vivo through an aptamer conjugated nanoparticle probes. The probe was synthesized by the AS1411 aptamer (MF-AS1411) which targeted nucleolin a cobalt-ferrite nanoparticle comprised core of fluorescent rhodamine and a silica-based shell. Then the probe was injected intravenously into nude mice bearing tumour xenografts and performed ^67^Ga radionuclide imaging and MRI. The SPECT image showed that this probe exhibited a better accumulation in tumour 24 h after injection than the control group. Furthermore, MRI images showed the AS1411 conjugated nanoparticle as black signal in tumours 24 h after injection, while there were no T2 negative signals in the control group. Finally, fluorescence images exhibited a higher signal in tumours of mice injected with aptamer conjugated nanoparticles compared to the control one. Taken together, these results proved that aptamers can deliver the nanoparticles to tumor tissue (Figure 8).

### 3.5. Multifunctional Molecular Imaging Agent

With multimodality imaging techniques clearly on the rise, the development over these new techniques has led to explosive growth in multimodal imaging agent researches [74, 75]. Since different imaging techniques can only detect their corresponding contrast agents, the patient who intends to perform multimodality imaging may need to inject different contrast mediums. This will increase the patient's economic burden and the additional stress on the blood clearance mechanisms. Moreover, it will expose him to side effect by the mutual interference between different contrast agents. Therefore, many researches are trying to design and develop a probe which can be detected by different imaging modalities so as to boost the clinical benefits of multimodality imaging. Due to their limited attachment points, small molecules are not suitable for multimodality imaging. On contrast, nanoparticles are attractive candidates for multimodal imaging probe [76–78]. They possess high surface areas to volume ratio, which allows multiple modifications to ligands and different imaging agents within the nanoparticles or on its surface. Recently, John et al. [77] described the use of a targeted multimodal protein-shell microsphere which contained iron oxide nanoparticles in their cores and conjugated with the RGD peptide ligand to enhance the imaging ability of ultrasound, MRI and MM-OCT. Also, Liu et al. [79] designed an iron oxide nanoparticle-embedded polymeric microbubble used for ultrasound imaging and MRI. Liang et al. [80] reported the synthesis and evaluation of streptavidin nanoparticle-based complexes which were functionalized with biotinylated anti-Her2 Herceptin antibody as multimodality imaging agents to detect tumor in a model via both SPECT system and IVSI fluorescence camera. Willmann et al. [81] prepared a polymeric contrast agent MBQDs - PLGA suited for optical and ultrasonic molecular imaging. In addition, they used PET to assess in vivo whole-body biodistribution of microbubbles functionalized with anti-VEGFR2 antibodies that were marked by the radiofluorination agent N-succinimidyl-4-[^18^F] fluorobenzoate (SFB) [82]. Hence, it can be seen that multimodal imaging agents are available, but their clinical application still need further research.

Apart from imaging agent and ligands, therapeutic drug or gene can also be incorporated in nanoparticles to construct theragnostic agent which have an important role in therapy. This agent enables observation of the extent of disease prior to therapeutic intervention. The ability to identify disease status and controlled delivery of drug using the same agent would help ensure that only potential responsive patients would be treated. Therefore, theragnostic agents show a promising prospective in therapy. Yan et al. [83] have synthesized a novel targeted drug-loaded microbubble functionalized with LyP-1, a breast tumor homing peptide, and evaluated its effect in vivo. Results of the study demonstrated that the LyP-1-coated PTX-loaded microbubble improved the antitumor efficacy markedly and had great potential in ultrasound-assisted breast cancer treatment.

## 4. Prospect

Molecular imaging technology truly enables dynamical, quantitative visualization of specific biochemical activity without trauma in vivo at cellular and molecular level. It has a direct impact on the modern and future medicine. In recent years, molecular imaging technology has seen certain progresses in the early diagnosis, curative effect monitoring of diseases, drug development, gene therapy, and other fields, but some key problems regarding theory, technology, and system, especially molecular imaging agents and imaging equipment, are not solved yet. The development of a new probe is not straightforward. Due to barriers in delivery, biological compatibility, and the diversity between species, there are only a few of clinical molecular imaging agents available currently. With the development of technology, it is expected that more advancement will be achieved in the area of molecular imaging agent including targeted molecular imaging agent and multifunctional molecular imaging agents. The application of nanoparticles has provided a platform for theranostic researches.

Additionally, although a number of molecular imaging instruments are available, all have their limitations. For instance, MRI is a useful clinical diagnostic tool, but it suffers from somewhat poor sensitivity. Therefore, more focus should be performed on refining and improving upon existing instrumentation and further efforts geared toward the combination of different modalities so as to make up the disadvantages of different instruments. Multimodality imaging has many advantages, but some problems still exist and need to be solved, such as the difficulty of designing a PET/MRI system suited for the entire body, cost increase, performance improvement, and multimodal contrast agent fusion.

Furthermore, as an emerging interdisciplinary science which brings together nuclear medicine, ultrasonic medicine, radiology, pharmacy, and materials science, the development of molecular imaging needs to strengthen interdisciplinary cooperation. Awareness of importance of multidisciplinary joint research should be strengthened.

The rapid expansion of molecular imaging application shows a promising prospect. Although overall the molecular imaging is still at the initial stage of development, we believe that within the support and cooperation from imaging experts and scholars, molecular imaging techniques would eventually realize clinical transformation.
